# Assessing the Impact of Metabolic Syndrome on Liver Outcomes in Methotrexate Users: A Retrospective Cohort Study

**DOI:** 10.3390/jcm14196799

**Published:** 2025-09-26

**Authors:** Yassine Kilani, Daniel Alejandro Gonzalez Mosquera, Kaila Fennell, Kamran Qureshi

**Affiliations:** 1 School of Medicine, Saint Louis University, St. Louis, MO 63104, USA; yassinekilanimd@gmail.com (Y.K.); kaila.fennell@health.slu.edu (K.F.); 2 New York Health + Hospitals|Lincoln, Weill Cornell Medical College Affiliate, Bronx, NY 10451, USA; gonzaled35@nychh.org

**Keywords:** hepatology, metabolic syndrome, drug-induced liver injury

## Abstract

**Background/Objectives**: Methotrexate (MTX) is associated with hepatotoxicity, but distinguishing MTX-induced liver injury from hepatic dysfunction due to metabolic syndrome (MetS) is challenging. This study investigates the incidence of hepatic outcomes in long-term MTX users with and without MetS and metabolic dysfunction-associated steatotic liver disease (MASLD) using real-world data. **Methods**: This cohort study used the TriNetX research network to identify U.S. adults (≥18 years) on MTX, excluding those with pre-existing liver injury. Patients were grouped by MetS status (MTX-MetS vs. controls) and further sub-stratified based on MASLD status. Propensity score matching (1:1) was adjusted for demographics, comorbidities, and treatment. The primary outcomes included hepatic-enzyme elevations, hyperbilirubinemia, prolonged INR, and clinically significant drug-induced liver injury (DILI) at 3-, 5-, and 10-year follow-up. **Results**: Among 324,219 MTX users, 59,733 MTX-MetS patients were propensity matched with 59,733 controls. MTX-MetS patients demonstrated increased 10-year odds of hepatic-enzyme elevations (aOR = 1.41; 95%CI: 1.38–1.46), hyperbilirubinemia (aOR = 1.40; 95%CI: 1.32–1.49), prolonged INR (aOR = 1.58; 95%CI: 1.49–1.67), clinically significant DILI (aOR = 1.49; 95%CI: 1.41–1.57), and liver cirrhosis (aOR = 1.48; 95%CI: 1.35–1.63) compared to the controls. Patients with and without MASLD showed similar hepatic-enzyme, bilirubin, and INR elevations, with higher odds of DILI with MASLD (aOR = 1.56; 95%CI: 1.28–1.89). **Conclusions**: This study highlights the increased liver injury risk in MTX users with MetS and MASLD. Further studies are needed to distinguish the effects of MTX and metabolic dysfunction on liver outcomes.

## 1. Introduction

Methotrexate (MTX) remains the first-line therapy for various chronic inflammatory and autoimmune conditions, including rheumatoid arthritis (RA), psoriatic arthritis (PA), and malignancies [1,2]. Despite its well-documented efficacy, concerns persist regarding methotrexate-induced liver injury, which affects approximately 20–22% of individuals treated with long-term MTX therapy [3]. A meta-analysis of RA and PA patients undergoing MTX treatment reported histologic progression of liver disease in 27% of cases, with 5% developing advanced liver fibrosis or cirrhosis (grade IIIB or IV) [4]. These findings underscore the necessity of routine hepatic surveillance in patients on prolonged MTX therapy to mitigate the risk of progressive liver damage and optimize long-term treatment safety [5,6,7].

Metabolic syndrome (MetS) is defined by a combination of criteria, including visceral obesity, hypertension, hypertriglyceridemia, reduced high-density lipoprotein (HDL) cholesterol, and impaired glucose metabolism. This constellation of metabolic derangements significantly increases susceptibility to hepatic disorders, most notably Metabolic Dysfunction–Associated Liver Disease (MASLD) and its progressive form, Metabolic Dysfunction-Associated Steatohepatitis (MASH), both of which may progress to hepatic fibrosis and cirrhosis [8,9,10,11]. MTX-induced hepatotoxicity is described as resembling steatohepatitis, similar to that seen in individuals with MetS, where it is termed MASH. This diagnostic ambiguity may prompt the discontinuation of MTX in favor of alternative immunomodulatory agents such as Leflunomide or Sulfasalazine [12,13,14,15]. However, distinguishing MTX-induced hepatic injury from other causes of liver dysfunction remains a significant clinical challenge, particularly in patients with underlying MetS. Although histological scores, such as the Roenigk Classification System, are useful for grading MTX-induced liver injury, they lack the specificity required to serve as definitive diagnostic tools for distinguishing MTX-induced liver injury from MetS/MASLD [16,17].

While the association between MTX and hepatotoxicity is well-documented, a critical gap remains in differentiating MTX-induced liver injury and hepatic dysfunction secondary to MetS [5,12,18]. Given the rising prevalence of MetS among patients receiving MTX, there is a significant risk of over diagnosing MTX hepatotoxicity individuals whose hepatic impairment may be attributable to MetS/MASLD. As highlighted by Cheema et al., liver injury in these patients is more frequently caused by MetS or other chronic liver conditions rather than MTX itself [19]. This diagnostic ambiguity may lead to unwarranted discontinuation of MTX, thereby compromising disease control, while simultaneously delaying appropriate interventions for MetS/MASLD.

Utilizing real-world data from a comprehensive national database, this study aims to test the hypothesis that MetS and MASLD are major drivers of incident hepatic dysfunction in MTX users, with a greater relative contribution compared to cumulative MTX exposure. To expand the existing body of evidence, we compared the incidence of abnormal liver biochemical profiles, clinically significant drug-induced liver injury (DILI), liver cirrhosis, hepatocellular carcinoma (HCC), and liver transplantation (LT) across study groups. We propose that the steatohepatitis observed in MTX-induced liver injury may represent MASH and is likely related to the presence of MetS. Consequently, we anticipate that liver injury and steatohepatitis will occur less frequently in long-term MTX users without MetS.

## 2. Materials and Methods

### 2.1. Data Source

This retrospective cohort study utilized the TriNetX Analytics Network Platform (Cambridge, MA, USA), a globally integrated federated research network that includes de-identified electronic health records (EHRs) from both U.S. and international healthcare organizations. For this analysis, data from 68 U.S. healthcare organizations (HCOs), representing 117 million patients, were included [20]. Using demographic and administrative data, this platform facilitates cohort selection and propensity score matching (PSM) application, allowing for comparative analysis of healthcare utilization and patient outcomes while accounting for potential confounders [20]. Data integrity is maintained through a rigorous quality assurance process enforced during EHR extraction, ensuring standardized formatting prior to database inclusion. This study employed publicly available de-identified data and is therefore exempt from Institutional Review Board (IRB) approval, as per the National Human Research Protections Advisory Committee guidelines [21]. The de-identification process, as stipulated by the Health Insurance Portability and Accountability Act (HIPAA) Privacy Rule, is performed at the network level by TriNetX experts [20].

### 2.2. Study Population and Variables

**Figure 1:**
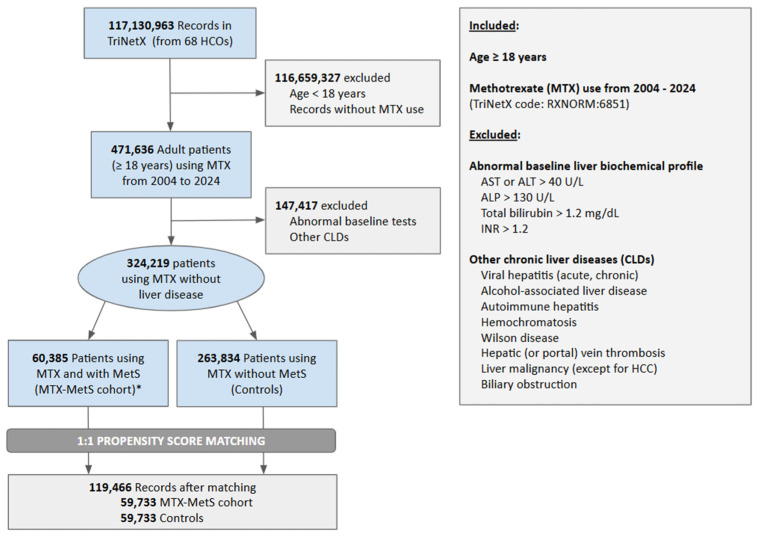
Flowchart of our study population. * The metabolic dysfunction-associated steatotic liver disease (MASLD) subgroup was derived from patients with metabolic syndrome (MetS), which may overestimate the overlap between MASLD and MetS. Patients with MASLD without MetS were not included in this analysis. Abbreviations: CLD, chronic liver disease; HCO, healthcare organization; MTX, methotrexate; MetS, metabolic syndrome.

A real-time search and analysis of the U.S. Collaborative Network using the TriNetX platform were conducted from 1 January 2004 to 1 January 2024. We analyzed records of adults (≥18 years) who received MTX, using TriNetX codes (RXNORM:6851) (Supplementary Table S1), with data extending retrospectively for up to 20 years prior to the designated analysis date [20]. Patients with prior occurrences of MTX use before this temporal threshold were excluded to ensure accurate incident case identification and reduce cohort selection bias. Using International Classification of Diseases, Tenth Revision Clinical Modification (ICD-10-CM) codes, patients with pre-existing chronic liver diseases (CLD) were excluded (e.g., viral hepatitis, alcohol-associated hepatitis, autoimmune hepatitis, hemochromatosis, Wilson disease, hepatic or portal vein thrombosis, malignancy except hepatocellular carcinoma (HCC), and biliary obstruction). We then stratified patients using MTX without other known pre-existing CLDs by the presence of MetS: those with MetS (e.g., MTX-MetS cohort) versus those without MetS (e.g., controls) (Figure 1). Furthermore, we stratified patients in the MTX-MetS cohort into two subgroups based on the presence of MASLD: MTX-MetS-MASLD and MTX-MetS-nonMASLD cohorts. MASLD was defined by the presence of ICD-10 codes for hepatic steatosis (K76.0) or metabolic-associated steatohepatitis (K75.81) in patients with MetS. This definition may overestimate the overlap between MASLD and MetS in this cohort. Both subgroups were compared to controls. The details of all ICD-10 and/or TriNetX codes used to define MetS and MASLD are provided in Supplementary Table S1.

In accordance with the criteria established by the National Cholesterol Education Program’s Adult Treatment Panel III (NCEP ATP III) criteria, MetS was defined as the presence of at least three of the following five criteria [22,23]: hypertriglyceridemia (≥150 mg/dL or treated for high triglycerides), reduced HDL cholesterol (<40 mg/dL), hypertension (systolic blood pressure ≥ 130 mmHg and/or diastolic blood pressure ≥ 85 mmHg or current antihypertensive treatment), impaired fasting glucose (≥100 mg/dL, or a prior diagnosis and/or treatment for type 2 diabetes mellitus), and obesity. The MetS criteria were identified using ICD-10-CM codes for diagnoses and TriNetX codes for blood pressure, fasting glucose, and HDL/LDL cholesterol, with values defined according to NCEP ATP III criteria, as detailed in Supplementary Table S1. Patients with at least three criteria were included in the analysis. Given the absence of waist circumference data within the TriNetX database, the Body Mass Index (BMI) was utilized as a substitute for abdominal (or visceral) obesity.

### 2.3. Patient and Hospital Characteristics

We retrieved data within the TriNetX database on age (mean with standard deviation), sex (male, female), race/ethnicity (non-Hispanic White, Black or African American, Hispanic or Latino), potential etiology for MTX use (e.g., rheumatoid arthritis (RA), juvenile idiopathic arthritis, psoriasis, connective tissue diseases, hematological malignancy, or any malignancy) [24], treatment with glucocorticoids, and social determinants of adverse health outcomes (SDHOs, defined by a set of validated ICD-10-CM codes known as ‘Z codes’ (Z55-Z65), which are endorsed by the American Hospital Association (AHA) Coding Clinic to capture social and economic factors that may lead to heightened social needs and impact various health and life outcomes) (Table 1) [25,26]. The Charlson Comorbidity Index components are included in Table 2. Following one-to-one (1:1) PSM based on these variables, we matched records of patients within the MTX-MetS cohort to the controls (Table 1 and Table 2). Similarly, in the subgroup analyses, we matched individuals within the MTX-MetS-MASLD and MTX-MetS-nonMASLD cohorts to the controls (Supplementary Tables S3 and S4). To ensure adequate exclusion of MetS criteria in our control group, we report the distribution of MetS criteria in the MTX-MetS cohort and controls in Supplementary Table S2.

### 2.4. Objectives and Outcomes

The primary outcomes included abnormal liver biochemical profiles and clinically significant DILI in the MTX-MetS cohort and its subgroups compared to the controls. Abnormal liver biochemical profiles were characterized based on 3 parameters: hepatic-enzyme elevations, hyperbilirubinemia, and a prolonged International Normalized Ratio (INR). Hepatic-enzyme elevations were captured using TriNetX codes for alanine aminotransferase (ALT) > 40 U/L (TNX:9044), aspartate aminotransferase (AST) > 40 U/L (TNX:9047), or alkaline phosphatase (ALP) > 130 U/L (TNX:9046). Similarly, hyperbilirubinemia was defined as serum bilirubin > 1.2 mg/dL (TNX:9050), and prolonged INR was identified by an INR > 1.2 (TNX:9032). Furthermore, clinically significant DILI was defined and captured using International Classification of Diseases, Tenth Revision Clinical Modification (ICD-10-CM) codes for toxic liver disease (K71.0, K71.1, K71.2, K71.6, K72.0, K72.9), along with TriNetX codes for elevated ALT or AST > 200 U/L, ALP > 200 U/L, serum bilirubin > 2.5 mg/dL, and International Normalized Ratio (INR) > 1.5 [27]. Secondary outcomes were identified using ICD-10-CM/ICD-10 Procedure Coding System (-PCS) codes and Current Procedural Terminology (CPT)/Healthcare Common Procedure Coding System (HCPCS) Codes, including liver cirrhosis, HCC (ICD-10-CM: C22.0); liver transplantation (LT) (ICD-10-PCS: 0FY00Z0, 0FY00Z1, 0FY00Z2; CPT: 47133, 47135, 47140); all-cause mortality (TriNetX: Deceased); hospitalizations (CPT: 1013659); and critical care (e.g., ICU) admissions (CPT: 1013729). Cirrhosis was defined using a composite of ICD-10-CM codes for liver cirrhosis (K71.7, K74.6), portal hypertension (K76.6), ascites (R18), esophageal varices (I85), spontaneous bacterial peritonitis (SBP) (K76.7), hepatic encephalopathy (K76.82), hepatorenal syndrome (HRS) (K76.7), and hepatopulmonary syndrome (K76.81) (Supplementary Table S1). Furthermore, we compared these outcomes in the subgroups (e.g., MTX-MetS-MASLD and MTX-MetS-nonMASLD cohorts) and controls (e.g., patients using MTX without MetS). Outcomes were observed at 3-year, 5-year, and 10-year follow-up (Table 3, Supplementary Tables S5–S7). Adjustments were made to account for potential confounders (e.g., demographics, SDHOs, comorbid conditions, and treatment). The reporting of this study adheres to the “Strengthening the Reporting of Observational Studies in Epidemiology” (STROBE) reporting guidelines, provided in Supplementary Table S8.

### 2.5. Statistical Analysis

Statistical analyses were conducted using the TriNetX Advanced Analytics Platform. Descriptive statistics for each group are presented as the mean ± standard deviation (SD) for continuous variables or frequency with proportion for categorical variables. To control for potential confounding variables, 1:1 PSM was performed for comparison between cohorts (e.g., MTX-MetS vs. controls) and for subgroup analyses (MTX-MetS-MASLD vs. controls; MTX-MetS-nonMASLD vs. controls). Propensity scores were generated, and patients were matched using a greedy nearest-neighbor algorithm with a caliper width of 0.1 standard deviations. Comparative analyses were conducted within matched groups. PSM was utilized to estimate the adjusted odds ratio (aOR) and 95% confidence interval (95%CI) of liver chemistry abnormalities, clinically significant DILI, and other outcomes adjusted for confounders. The baseline characteristics were compared using *t*-tests for continuous variables and Chi-square tests for categorical variables. A two-sided *p*-value < 0.05 was pre-defined as the threshold for statistical significance. A Bonferroni correction was applied to all univariate tests to account for multiple comparisons, resulting in an adjusted significance level of *p* < 0.0019.

## 3. Results

### 3.1. Study Population

A total of 117,130,963 records were found within the U.S. Collaborative Network, and 471,174 adult patients (≥18 years) had a history of MTX use prior to 1 January 2024. Patients with pre-existing abnormal liver biochemical profiles or other CLDs were excluded, yielding a final cohort of 324,219 MTX users without evidence of pre-existing liver disease. Within this cohort, 60,385 individuals (19%) fulfilled the diagnostic criteria for MetS, thereby comprising the MTX-MetS cohort, whereas the remaining 263,834 individuals (81%) served as the control group (Figure 1). A total of 652 records from the MTX-MetS cohort and 204,101 control records were excluded due to insufficient similarity, resulting in two propensity-score-matched cohorts comprising 119,466 records evenly distributed between the MTX-MetS cohort (n = 59,733) and controls (n = 59,733) (Figure 1). Additionally, 1:1 PSM was conducted to match patients within the MTX-MetS-MASLD (n = 4654) and MTX-MetS-nonMASLD (n = 54,638) cohorts to the control subjects (n = 263,834), resulting in matched pairs of 4625 patients for the MTX-MetS-MASLD vs. controls comparison, and 54,638 patients for MTX-MetS-nonMASLD vs. controls analysis.

### 3.2. Patient Characteristics

Among the unmatched samples, individuals in the MTX-MetS cohort (n = 60,385) were significantly older, and exhibited a higher prevalence of male sex, Black or African American race, rheumatoid arthritis, psoriasis, connective tissue disorders, malignancy, Charlson comorbidities, glucocorticoid therapy, and SDHO compared to the control group (n = 54,001) (*p* < 0.001) (Table 1 and Table 2). A similar distribution of baseline characteristics was observed in subgroups with and without MASLD (Supplementary Tables S3 and S4). To mitigate the influence of confounding variables on the study outcomes, PSM was employed, to ensure a well-balanced distribution of covariates and to enhance the comparability between the treatment and control groups. Additionally, a comprehensive assessment of MetS components showed that individuals within the control cohort had lower rates of hypertension (18% vs. 39%), BMI (mean: 29.6 ± 7.6 vs. 33.2 ± 7.5 kg/m^2^), TG (mean: 123 ± 87.4 mg/dL vs. 147 ± 103 mg/dL), fasting serum glucose (mean: 105 ± 37.3 vs. 120 ± 52.8 mg/dL) and antidiabetic medications use (8% vs. 26%). Additionally, the control group demonstrated a higher mean HDL (55.9 ± 18.6 vs. 50.6 ±18) compared to the MTX-MetS cohort (Supplementary Table S2).

### 3.3. Laboratory Abnormalities

The liver-related outcomes among patients receiving MTX with/without MetS are summarized in Table 3 and Supplementary Tables S5–S7. Among MTX users, the presence of MetS (n = 59,733) was associated with significantly increased odds of hepatic-enzyme elevations at 3-year (13% vs. 10%; aOR = 1.34; 95%CI: 1.30–1.93), 5-year (17% vs. 13%; aOR = 1.38; 95%CI: 1.34–1.43), and 10-year (21% vs. 15%; aOR = 1.41; 95%CI: 1.38–1.46) follow-up. Similarly, patients in the MTX-MetS cohort demonstrated a significantly higher likelihood of developing hyperbilirubinemia (4% vs. 3%; aOR = 1.40; 95%CI: 1.32–1.49) and prolonged INR (5% vs. 3%; aOR = 1.58; 95%CI: 1.49–1.67) over the 10-year follow-up period (Table 3, Supplementary Table S7).

Subgroup analyses revealed that patients with and without MASLD exhibited comparable elevations in hepatic enzymes, serum bilirubin, and INR over the 10-year follow-up period relative to the controls (Table 3, Supplementary Tables S5–S7).

### 3.4. Liver-Related Outcomes

Furthermore, patients in the MTX-MetS cohort had significantly increased odds of developing clinically significant DILI at 3-year (3% vs. 2%; aOR = 1.45; 95%CI: 1.35–1.56), 5-year (4% vs. 3%; aOR = 1.38; 95%CI: 1.36–1.55), and 10-year (5% vs. 4%; aOR = 1.49; 95%CI: 1.41–1.57) follow-up. This association was more pronounced in patients with MASLD (MTX-MetS-MASLD) (6% vs. 4%; aOR = 1.56; 95%CI: 1.28–1.89), whereas those without MASLD (MTX-MetS-nonMASLD) had a comparable increase in the 10-year odds of clinically significant DILI (5% vs. 4%; aOR = 1.41; 95%CI: 1.33–1.50) compared to the controls (Table 3, Supplementary Tables S5–S7).

Lastly, patients in the MTX-MetS cohort exhibited significantly higher odds of liver cirrhosis at 10-year follow-up (2% vs. 1%; aOR = 1.48; 95%CI: 1.35–1.63). This association was more pronounced in those with MASLD (4% vs. 2%; aOR = 2.21; 95%CI: 1.72–2.84), while patients without MASLD (MTX-MetS-nonMASLD) had a comparatively smaller increase in the 10-year odds of clinically significant DILI (1.4% vs. 1.2%; aOR = 1.22; 95%CI: 1.10–1.36) relative to the controls (Table 3, Supplementary Tables S5–S7).

### 3.5. Mortality and Healthcare Resource Utilization

The mortality and healthcare resource utilization among patients receiving MTX with/without MetS are detailed in Table 3 and Supplementary Tables S5–S7. In MTX users, the presence of MetS (n = 59,733) was associated with a modest increase in all-cause mortality (aOR = 1.13; 95%CI: 1.08–1.18). However, a significant increase in all-cause hospitalizations (aOR = 1.43; 95%CI: 1.39–1.47) and ICU admissions (aOR = 1.60; 95%CI: 1.52–1.69) was observed at 10 year follow-up compared to the matched controls (n = 59,733) (Table 3). These findings remained consistent across subgroups with and without MASLD (Table 3, Supplementary Tables S5–S7).

## 4. Discussion

This cohort study investigated the impact of MetS on hepatic outcomes in MTX users, using real-world data from a large U.S.-based health network. Among MTX users, those with MetS had a higher likelihood of hepatic injury compared to those without, as evidenced by a higher incidence of hepatic-enzyme elevations, DILI, and cirrhosis over a 10-year period. These associations were exacerbated in patients with concurrent MetS and MASLD, underscoring the predominant role of metabolic dysfunction in hepatic impairment within this cohort. These results challenge the belief that hepatic dysfunction primarily stems from direct MTX hepatotoxicity and instead suggest that metabolic derangements serve as key drivers of liver injury in this population [5,6,7,12]. However, given this study’s retrospective design and reliance on coding-based definitions, these findings should be interpreted with caution. While the associations observed are strong, they do not establish causality, and prospective studies are required to confirm these relationships.

MTX remains a well utilized therapy for autoimmune, inflammatory, dermatologic, and hematologic diseases, yet concerns regarding hepatotoxicity often prompt close monitoring and, in some cases, permanent drug discontinuation [1,13,15,23]. While MTX is historically described to induce liver injury, our analysis suggests that metabolic dysfunction plays a more significant role in hepatic injury and long-term outcomes than MTX exposure alone in those using MTX for various conditions. MTX is known to cause hepatic injury through direct mitochondrial toxicity, oxidative stress, and inhibition of hepatic folate metabolism, leading to hepatocellular apoptosis and fibrosis [1,2,12]. Interestingly, similar mechanisms—mitochondrial dysfunction, oxidative stress, and altered lipid metabolism—also contribute to MASLD-related liver injury, creating a confounding effect in patients with MetS [10,11]. These data highlight the need to distinguish between MTX hepatotoxicity and liver injury attributable to MetS and MASLD. As previously highlighted by Cheema et al., histopathological features traditionally ascribed to MTX-induced hepatotoxicity may, in many cases, reflect the concomitant presence of MetS/MASLD [19]. This overlap raises critical questions regarding the validity of “MTX-induced” liver injury as a distinct pathological entity, suggesting that metabolic dysfunction may be the primary driver of hepatic injury in these patients rather than direct MTX toxicity [16,17,18]. In our study, MetS was present in 18.6% of all MTX users, and among these, 7.7% had MASLD, translating to an overall MASLD prevalence of ~1.4% among all MTX users in this study. Furthermore, the primary indications for MTX use in this study were connective tissue diseases (10.2%), psoriasis (8.9%), and RA (seropositive 7.1%, seronegative 2.7%), all of which involve chronic systemic inflammation that may contribute to liver injury independently of MTX exposure in the control group [5,12]. Glucocorticoids, used in 52.9% of the controls, were accounted for in PSM; however, their concomitant use may have further contributed to hepatic injury [24].

This study leverages a large-scale real-world dataset (TriNetX) encompassing over 324,000 MTX users, allowing for robust subgroup analyses and enhanced generalizability [20]. Rigorous exclusion criteria minimized confounding by pre-existing CLDs (e.g., viral hepatitis, alcohol-associated liver disease), ensuring an accurate assessment of MTX-related liver injury [5,12]. Furthermore, baseline liver function abnormalities were excluded to minimize selection bias and capture new-onset dysfunction. PSM adjusted for key confounders, including comorbidities, medication use (e.g., glucocorticoids), and social determinants of health, reinforcing the internal validity of our results [24,25,26]. Subgroup analysis highlighted MASLD as a stronger driver of liver injury than MTX alone [12]. Furthermore, longitudinal follow-up (3-, 5-, and 10-year) provided a clinically relevant perspective, while real-world data enhanced the applicability of our results to clinical practice, informing MTX management based on metabolic risk.

Despite its strengths, this study has certain limitations. Firstly, MASLD was identified using ICD-10 codes only among patients with pre-existing MetS; its presence in the control group (without MetS) was not assessed and is likely underestimated. Consequently, our analysis may overestimate the association and overlap between MetS and MASLD in the MTX-exposed population, introducing potential detection bias. Secondly, although patients with other CLDs were excluded, concomitant use of other hepatotoxic agents (e.g., antifungals, anticonvulsants, and other immunosuppressants) could not be accounted for and may have influenced the observed associations [5,12,23]. The lack of cumulative MTX dose and duration data in the TriNetX database precludes assessment of dose-dependent hepatotoxicity [6,7]. Another limitation is the use of BMI as a surrogate for obesity instead of waist circumference, which is a more precise marker for visceral adiposity and metabolic risk [23]. While BMI is widely available and convenient for large datasets, it is a flawed surrogate for central adiposity, and this substitution may attenuate the observed associations between obesity and hepatic risk. This study used carefully selected ICD-10 codes; however, their use can affect accuracy, as diagnoses and laboratory data may vary across institutions. For instance, identification of clinically significant DILI relied on ICD-10 and TriNetX codes aligning with Fontana criteria, which may lack sensitivity for subclinical DILI and introduce coding inaccuracies [27]. Future studies using standardized tools such as RUCAM are needed to validate MTX-DILI and clarify the contribution of concomitant MASLD [27]. Unmeasured confounders may exist despite PSM, and the reliance on EHR-based coding introduces the possibility of misclassification or missing data. In fact, adjustment for systemic inflammation—a critical confounder—was not feasible due to the absence of routinely captured markers such as CRP or ESR, potentially resulting in residual confounding [5,12]. Furthermore, the use of medications that could potentially modify metabolic risk, such as GLP-1 receptor agonists, was not captured, representing both a limitation and a potential area for future prospective studies to determine whether these therapies exert a protective effect against MTX-associated liver injury in high-risk populations [10]. The retrospective design of this study prevents us from drawing definitive conclusions regarding causality. While strong associations were identified between MTX use, MetS, and MASLD, these results should be interpreted with caution as they remain observational and hypothesis-generating rather than causal. Additionally, the absence of histologic confirmation via liver biopsy or non-invasive fibrosis assessments (e.g., FibroScan, FIB-4) complicated differentiation between MTX-induced hepatotoxicity and metabolic liver disease [16,17]. As a result, we cannot definitively separate MTX-related liver injury from MASLD-related damage, and some cases labeled as MTX hepatotoxicity may in fact reflect underlying MASLD. This overlap is especially important given the histological similarities between the two conditions. Lastly, genetic and pharmacogenomic data, including MTHFR polymorphisms, were unavailable, limiting our ability to assess for individual susceptibility to MTX hepatotoxicity [24].

These findings highlight the need for a more personalized approach to liver monitoring in MTX users. The conventional practice of routine liver function tests for all MTX patients may lead to unnecessary drug discontinuation in those misclassified as having MTX hepatotoxicity, when underlying metabolic dysfunction could be driving liver injury. Given that MetS was present in 18.6% of all MTX users, with 7.7% of MTX-MetS patients having MASLD, liver surveillance should be tailored to prioritize those with metabolic risk factors rather than applying a uniform approach to all MTX users [8,9,10,11]. Future guidelines should emphasize metabolic risk stratification in MTX hepatotoxicity assessments, ensuring more accurate clinical decision-making and better long-term patient outcomes.

## 5. Conclusions

In conclusion, this study demonstrates a strong association between MetS and MASLD, with increased liver injury risk in MTX users, suggesting that metabolic dysfunction may play a more significant role in hepatic outcomes than MTX hepatotoxicity alone. This challenges the conventional attribution of liver injury solely to MTX toxicity, and contributes to the shift in clinical perception regarding MTX toxicity. While prospective studies are needed to confirm causality and refine risk stratification, this study provides a compelling evidence base for moving beyond cumulative dosing to more personalized, metabolic-focused, and intensified hepatic surveillance strategies in patients with MetS and/or MASLD using MTX.

## Figures and Tables

**Table 1:** Baseline characteristics when comparing individuals using methotrexate (MTX) and with a prior history of metabolic syndrome (MetS) (MTX-MetS cohort, n = 60,385) compared to those without MetS (Controls, n = 263,834). Propensity score matching resulted in 59,733 matched pairs.

	Before Propensity Matching			After Propensity Matching		
Baseline Characteristics	MTX-MetS	Controls	*p*	MTX-MetS	Controls	*p*
Age (Mean ± SD)	60.5 ± 13.7	53.4 ± 18.0	<0.001	60.5 ± 13.7	60.7 ± 13.8	0.002
Sex: Female	68.6% (41,007)	73.8% (191,128)	0.113	68.6% (41,006)	68.8% (41,068)	0.699
Race/Ethnicity						
Non-Hispanic White	64.1% (38,302)	65.7% (170,143)	0.032	64.1% (38,302)	64.2% (38,323)	0.899
Black or African American	17.2% (10,285)	13.0% (33,748)	0.117	17.2% (10,284)	17.2% (10,275)	0.945
Hispanic or Latino	7.3% (4354)	7.6% (19,804)	0.013	7.3% (4354)	7.2% (4307)	0.600
Etiology for MTX Use						
Seropositive RA	7.1% (4244)	4.9% (12,570)	<0.001	7.1% (4243)	6.7% (4018)	0.010
Seronegative RA	2.7% (1642)	1.8% (4535)	<0.001	2.7% (1642)	2.5% (1479)	0.003
Juvenile idiopathic arthritis	0.4% (261)	1.2% (2982)	<0.001	0.4% (261)	0.3% (204)	0.008
Psoriasis	8.9% (5306)	6.4% (16,587)	<0.001	8.9% (5306)	8.6% (5143)	0.095
Connective tissue diseases	10.2% (6114)	8.7% (22,509)	<0.001	10.2% (6114)	10.0% (5990)	0.234
Hematopoietic malignancy	2.3% (1394)	1.6% (4200)	<0.001	2.3% (1394)	2.3% (1360)	0.512
Any malignancy	21.6% (12,918)	14.6% (37,717)	<0.001	21.6% (12,917)	21.6% (12,926)	0.950
Glucocorticoid therapy	53.4% (31,876)	40.4% (104,724)	<0.001	53.4% (31,875)	52.9% (31,582)	0.089
SDHOs	1.4% (846)	1.0% (2462)	<0.001	1.4% (846)	1.4% (819)	0.512

Abbreviations: BMI, Body Mass Index; MetS, metabolic syndrome; MTX, methotrexate; RA, rheumatoid arthritis; SD, standard deviation; SDHOs, social determinants of adverse health outcomes.

**Table 2:** Charlson comorbidities in patients when comparing individuals using methotrexate (MTX) and with a prior history of metabolic syndrome (MetS) (MTX-MetS cohort, n = 60,385) compared to those without MetS (Controls, n = 263,834). Propensity matching resulted in 59,733 matched pairs. International Classification of Diseases, Tenth Revision Clinical Modification (ICD-10-CM) codes were used for the Charlson Comorbidity Index components. Patients with other liver diseases were excluded from the analysis.

		Before Propensity Matching			After Propensity Matching		
Charlson Comorbidities	ICD-10 CM CODES	MTX-MetSα	Controls	*p*	MTX-MetS	Controls	*p*
HIV/AIDS	B20	0.1% (73)	0.1% (211)	0.003	0.1% (73)	0.1% (68)	0.674
Cerebrovascular diseases	I60-I69	5.9% (3511)	2.5% (6565)	<0.001	5.9% (3510)	5.9% (3526)	0.844
Chronicpulmonary disease	J40-J4A	16.0% (9538)	9.3% (24,038)	<0.001	16.0% (9537)	15.9% (9481)	0.658
Congestive heart failure	I50	3.9% (2352)	1.4% (3752)	<0.001	3.9% (2351)	3.8% (2252)	0.137
Ischemic heart diseases	I20-I25	10.9% (6518)	4.1% (10,730)	<0.001	10.9% (6517)	10.8% (6465)	0.629
Dementia	F01-F09	1.5% (916)	0.9% (2258)	<0.001	1.5% (916)	1.5% (893)	0.586
Peptic ulcer disease	K25	0.7% (410)	0.4% (1025)	<0.001	0.7% (410)	0.7% (397)	0.646
Peripheral vascular disease	I70-I79	8.3% (4951)	4.8% (12,472)	<0.001	8.3% (4950)	8.2% (4878)	0.448
Paralytic syndromes *	G80-G83	0.8% (503)	0.4% (1039)	<0.001	0.8% (503)	0.8% (476)	0.386
Chronic kidney disease	N18	4.7% (2812)	1.7% (4489)	<0.001	4.7% (2811)	4.5% (2684)	0.079
Connective tissue diseases	M30-M36	10.2% (6114)	8.7% (22,509)	<0.001	10.2% (6114)	10.0% (5990)	0.234
Neoplasms	C00-D49	21.6% (12,918)	14.6% (37,717)	<0.001	21.6% (12,917)	21.6% (12,926)	0.950

Abbreviations: CLD, chronic liver disease; HIV/AIDS, Human Immunodeficiency Virus/Acquired Immunodeficiency Syndrome; MTX, methotrexate; MetS, metabolic syndrome. * Hemiplegia, paraplegia, and other paralytic syndromes.

**Table 3:** Propensity score matching analysis comparing the 3-, 5-, and 10-year outcomes of patients on methotrexate (MTX) with metabolic syndrome (MetS) (MTX-MetS cohort, n = 59,733) compared to those without MetS (Controls, n = 59,733). Due to scarcity of cases, we were unable to run the analysis for LT and HCC incidence (≤10 cases in both groups). Patients with pre-inclusion outcomes were excluded from the analysis.

	Incidence % (N) MTX-MetS vs. Controls			PSM Analysis (10-Year Outcomes) aOR * [95%CI]	
Outcomes	3-Year	5-Year	10-Year	MTX-MetS vs. Controls **	MTX-MetS-MASLD vs. Controls **
Laboratory abnormalities					
Hepatic-enzyme elevations ^1^	13.3% (7768) vs. 10.3% (6125)	17.0% (9891) vs. 12.9% (7674)	20.7% (12,051) vs. 15.5% (9255)	1.41 [1.38–1.46]	1.31 [1.17–1.46]
Hyperbilirubinemia ^2^	2.5% (1460) vs. 1.8% (1046)	3.2% (1932) vs. 2.3% (1384)	4.1% (2470) vs. 3.0% (1787)	1.40 [1.32–1.49]	1.57 [1.27–1.95]
Prolonged INR ^3^	3.2% (1883) vs. 2.1% (1227)	4.2% (2500) vs. 2.7% (1636)	5.4% (3216) vs. 3.5% (2087)	1.58 [1.49–1.67]	1.52 [1.24–1.88]
Liver-related Outcomes					
Clinically significant DILI ^4^	3.1% (1835) vs. 2.1% (1282)	4.1% (2441) vs. 2.9% (1711)	5.3% (3175) vs. 3.7% (2181)	1.49 [1.41–1.57]	1.56 [1.28–1.89]
Liver cirrhosis	1.1% (634) vs. 0.8% (453)	1.4% (857) vs. 1.0% (576)	1.9% (1102) vs. 1.3% (748)	1.48 [1.35–1.63]	2.21 [1.72–2.84]
Other Clinical Outcomes					
All-cause mortality	4.5% (2652) vs. 4.0% (2397)	6.2% (3715) vs. 5.6% (3314)	8.5% (5045) vs. 7.6% (4514)	1.13 [1.08–1.18]	0.88 [0.75–1.03]
All cause hospitalizations	10.5% (5602) vs. 7.4% (4015)	13.6% (7247) vs. 9.6% (5206)	17.3% (9171) vs. 12.1% (6550)	1.43 [1.39–1.47]	1.36 [1.20–1.54]
All-cause ICU admissions	3.6% (2118) vs. 2.3% (1360)	4.8% (2819) vs. 3.1% (1826)	6.4% (3745) vs. 4.1% (2401)	1.60 [1.52–1.69]	1.46 [1.22–1.75]

Abbreviations: aOR, adjusted odds ratio; DILI, drug-induced liver injury; INR, International Normalized Ratio; MTX, methotrexate; MetS, metabolic syndrome; PSM, propensity score matching. * Adjusted for demographics, comorbid conditions, and treatment. ^1^: Hepatic-enzyme elevation is defined as elevated alanine aminotransferase (ALT) > 40 U/L, aspartate aminotransferase (AST) > 40 U/L, or alkaline phosphatase (ALP) > 130 U/L. ^2^: Hyperbilirubinemia is defined as a serum bilirubin level > 1.2 mg/dL. ^3^: Prolonged INR is defined as an INR > 1.2. ^4^: Clinically significant DILI is defined as ALT or AST > 200 U/L, ALP > 200 U/L, serum bilirubin > 2.5 mg/dL, and International Normalized Ratio (INR) > 1.5 or International Classification of Diseases, Tenth Revision Clinical Modification (ICD-10-CM) codes for toxic liver injury or acute liver failure. **: Controls are defined as patients without metabolic syndrome (MetS) using methotrexate (MTX).

## Data Availability

The data that support the findings of this study were obtained from TriNetX. Due to licensing restrictions and patient privacy concerns, these data are not publicly available. Access to TriNetX data is limited to institutions with a license. Researchers interested in accessing similar data can do so by contacting TriNetX (https://www.trinetx.com) for more information on data access and collaboration opportunities.

## Supplementary Materials

The following supporting information can be downloaded at: https://www.mdpi.com/article/10.3390/jcm14196799/s1, Table S1: International Classification of Diseases, Tenth Revision, Clinical Modification/Procedure Coding System (ICD-10-CM/-PCS) codes, Current Procedural Terminology (CPT)/Healthcare Common Procedure Coding System (HCPCS) Codes, and TriNetX codes for the outcomes in our study population; Table S2: Baseline characteristics of our unmatched samples, showing adequate exclusion of metabolic syndrome components in the control group.; Table S3: Baseline characteristics when comparing individuals using methotrexate (MTX) and with a prior history of metabolic syndrome (MetS) and Metabolic dysfunction—Associated Liver Disease (MASLD) (MTX-MetS-MASLD cohort, n = 4654) compared to those without MetS (Controls, n = 263,834). Propensity score matching resulted in 4625 matched pairs; Table S4: Baseline characteristics when comparing individuals using methotrexate (MTX) and with a prior history of metabolic syndrome (MetS) without Metabolic dysfunction—Associated Liver Disease (MASLD) (MTX-MetS-nonMASLD cohort, n = 54,638) compared to those without MetS (Controls, n = 263,834). Propensity score matching resulted in 54,638 matched pairs; Table S5: Propensity score matching analysis comparing the 3-, 5-, and 10-year outcomes of patients on methotrexate (MTX), and with a history of metabolic syndrome (MetS) and Metabolic dysfunction—Associated Liver Disease (MASLD) (MTX-MetS-MASLD cohort, n = 4654 compared to those without MetS (Controls, n = 263,834). Due to scarcity of cases, we were unable to run the analysis for LT incidence (≤ 10 cases in both groups). Patients with pre-inclusion outcomes were excluded from the analysis; Table S6: Propensity score matching analysis comparing the 3-, 5-, and 10-year outcomes of patients on methotrexate (MTX) with metabolic syndrome (MetS) and without Metabolic dysfunction—Associated Liver Disease (MASLD) (MTX-MetS-nonMASLD cohort, n = 27,336) compared to those without MetS (Controls, n = 27,336). Due to scarcity of cases, we were unable to run the analysis for LT incidence (≤ 10 cases in both groups). Patients with pre-inclusion outcomes were excluded from the analysis; Table S7: Propensity score matching analysis comparing the 3-, 5-, and 10-year outcomes of patients on methotrexate (MTX) with metabolic syndrome (MetS) compared to those without MetS. Due to scarcity of cases, we were unable to run the analysis for LT and HCC incidence (≤ 10 cases in both groups). Patients with pre-inclusion outcomes were excluded from the analysis; Table S8: STROBE Statement.

## Abbreviations

The following abbreviations are used in this manuscript:

DILI	Drug-induced liver injury
MASLD	Metabolic dysfunction-associated steatotic liver disease
MetS	Metabolic syndrome
MTX	Methotrexate
PA	Psoriatic arthritis
RA	Rheumatoid arthritis
