# Supplementary material for: Assessing the Impact of Metabolic Syndrome on Liver Outcomes in Methotrexate Users: A Retrospective Cohort Study
Source: J Clin Med. 2025 Sep 26;14(19):6799. doi: 10.3390/jcm14196799 (PMC12524370; doi:10.3390/jcm14196799)
Supplement: Supplementary file 1 [file jcm-14-06799-s001.zip › jcm-3817792-supplementary.pdf]

Supplementary Table 1. International Classification of Diseases, Tenth Revision, Clinical Modification / Procedure Coding System (ICD-10-CM/ -PCS) codes, Current Procedural Terminology (CPT)/Healthcare Common Procedure Coding System (HCPCS) Codes, and TriNetX codes for the outcomes in our study population

	**Codes and nomenclature**
**INCLUSION CRITERIA**	
**Methotrexate (MTX)**	TriNetX: RXNORM:6851
**METABOLIC SYNDROME**	
**Obesity** (e.g. body mass index ≥ 30 kg/m^2)	TriNetX: TNX:9083
**Hypertension** (e.g. blood pressure ≥ 130/85 mmHg or treated with antihypertensives)	ICD-10-CM:<br>I10 - Essential (primary) hypertension<br><br>TriNetX:<br>TNX:9085 - systolic BP<br>TNX:9086 - diastolic BP<br>NLM:ATC:C09 - agents acting on the renin angiotensin system<br>NLM:ATC:C03A - thiazide diuretics<br>NLM:ATC:C08C - calcium channel blockers (vascular)<br>NLM:ATC:C02 - other antihypertensives
**Hypertriglyceridemia** (e.g. serum triglyceride ≥ 150 mg/dL or current treatment for hypertriglyceridemia)	ICD-10-CM:<br>E78.1 - Pure hyperglyceridemia<br><br>TriNetX:<br>TNX:9004 – serum triglycerides<br>NLM:ATC: C10AA - statins<br>NLM:ATC: C10AB - fibrates<br>RXNORM : 7393 - niacin<br>RXNORM : 4301 - omega-3 fatty acids
**Reduced High-density lipoprotein (HDL) cholesterol** reduced (e.g. HDL < 40 mg/dL)	TriNetX: TNX:9001
**Impaired fasting glucose** (e.g. fasting serum glucose ≥ 100 mg/dL or current treatment for diabetes mellitus)	ICD-10-CM:<br>E11 - Type 2 diabetes mellitus<br><br>TriNetX:

	LNC:1558-6 - fasting serum glucose ≥ 100 mg/dL<br>ATC:A10 - drugs used in diabetes
**SUBGROUPS**	
**Metabolic dysfunction-associated steatotic liver disease (MASLD)**	ICD-10-CM: K76.0, K75.81
**EXCLUSION CRITERIA**	
**Alcohol-associated liver disease**	ICD-10-CM: K70
**Viral hepatitis**	ICD-10-CM: B15-B19
**Autoimmune hepatitis**	ICD-10-CM: K75.4
**Wilson's disease**	ICD-10-CM: E83.01
**Hemochromatosis**	ICD-10-CM: E83.11
**Portal or hepatic vein thrombosis**	ICD-10-CM: I81, I82.0
**Warfarin**	TriNetX: RXNORM:11289
**Obstruction of bile duct**	ICD-10-CM: K83.1
**Liver malignancy** (except hepatocellular carcinoma)	ICD-10-CM:<br>C22.1 - Intrahepatic bile duct carcinoma<br>C22.2 - Hepatoblastoma<br>C22.3 - Angiosarcoma of liver<br>C22.4 - Other sarcomas of liver<br>C22.7 - Other specified carcinomas of liver<br>C22.8 - Malignant neoplasm of liver, primary, unspecified as to type<br>C22.9 - Malignant neoplasm of liver, not specified as primary or secondary

**Abnormal baseline liver biochemical profile** (e.g. Alanine aminotransferase (ALT) > 40 U/L, aspartate aminotransferase (AST) > 40 U/L, alkaline phosphatase (ALP) > 130 U/L, total bilirubin > 1.2 mg/dL, International Normalized Ratio (INR) > 1.2)	TriNetX:<br>TNX:9044, TNX:9047, TNX:9046, TNX:9050, TNX:9032
**TREATMENT**	
**Glucocorticoids**	TriNetX: VA:HS051
**OUTCOMES**	
**Hepatic enzyme elevations** (e.g. ALT > 40 U/L, AST > 40 U/L, ALP > 130 U/L)	TriNetX:<br>TNX:9044, TNX:9047, TNX:9046
**Hyperbilirubinemia** (e.g. total bilirubin > 1.2 mg/dL)	TriNetX: TNX:9050
**Prolonged INR** (e.g. INR > 1.2)	TriNetX: TNX:9032
**DILI – Clinically significant** (e.g. ALT or AST > 200 U/L, ALP > 200 U/L, TB > 2.5 mg/dL, INR > 1.5 or ICD-10-CM codes for toxic liver injury, or acute liver failure)	ICD-10-CM:<br>K71.0 - Toxic liver disease with cholestasis<br>K71.1 - Toxic liver disease with hepatic necrosis<br>K71.2 - Toxic liver disease with acute hepatitis<br>K71.6 - Toxic liver disease with hepatitis, not elsewhere classified<br>K72.0 - Acute and subacute hepatic failure<br>K72.9 - Hepatic failure, unspecified<br><br>TriNetX:<br>TNX:9044, TNX:9047, TNX:9046, TNX:9050, TNX:9032
**Liver cirrhosis**	ICD-10-CM:<br>K71.7 - Toxic liver disease with fibrosis and cirrhosis of liver<br>K74.6 - Other and unspecified cirrhosis of liver<br>K76.6 - Portal hypertension<br>R18 - Ascites<br>I85 - Esophageal varices<br>K65.2 - Spontaneous bacterial peritonitis<br>K76.7 - Hepatorenal syndrome<br>K76.81 - Hepatopulmonary syndrome<br>K76.82 - Hepatic encephalopathy

**Hepatocellular carcinoma**	ICD-10-CM:<br>C22.0 - Liver cell carcinoma
**Liver transplantation**	ICD-10-PCS:<br>0FY00Z0 - Transplantation of Liver, Allogeneic, Open Approach<br>0FY00Z1 - Transplantation of Liver, Syngeneic, Open Approach<br>0FY00Z2 - Transplantation of Liver, Zooplastic, Open Approach<br><br>CPT:<br>47133 - Donor hepatectomy (including cold preservation), from cadaver donor<br>47135 - Liver allotransplantation, orthotopic, partial or whole, from cadaver or living donor, any age<br>47140 - Donor hepatectomy (including cold preservation), from living donor; left lateral segment only (segments II and III)
**All-cause mortality**	TriNetX: Deceased
**All-cause hospitalization**	CPT: 1013659 - Hospital Inpatient and Observation Care Services
**Intensive care unit (ICU) admission**	CPT: 1013729 - Critical Care Services

Abbreviations: MTX, methotrexate, MetS, metabolic syndrome.

Supplemental Table 2. Baseline characteristics of our unmatched samples, showing adequate exclusion of metabolic syndrome components in the control group.

	ICD-10 CM codes	MTX-MetS	Controls
**Comorbid conditions**			
**Type 2 diabetes mellitus**	E11	22% (13,342)	7% (15,687)
**Essential hypertension**	I10	39% (22999)	18% (41789)
**Hypertriglyceridemia**	E78.1	1% (854)	0% (1161)
**Clinical/laboratory values (Mean ± SD)**			
**Body mass index (BMI) (kg/m^2)**	TNX:9083	33.2 ± 7.5	29.6 ± 7.6
**HDL cholesterol (mg/dL)**	TNX:9001	50.6 ±18	55.9 ± 18.6

**Triglycerides (mg/dL)**	TNX:9004	147 ± 103	123 ± 87.4
**Fasting Serum Glucose (mg/dL)**	TNX:9025	120 ± 52.8	105 ± 37.3
**Treatment**			
**Antidiabetic medications**	NLM:ATC:A10	26% (15,816)	8% (17,986)
**Antihypertensive treatment**			
**Drugs Acting on the renin angiotensin system**	NLM:ATC:C09	33% (19,443)	13% (30,862)
**Thiazide diuretics**	NLM:ATC:C03	20% (11,902)	8% (19,999)
**Selective calcium channel blockers (vascular)**	NLM:ATC:C08C	16% (9,330)	7% (16,522)
**Other antihypertensives**	NLM:ATC:C02	9% (5,198)	4% (9,058)
**Lipid lowering medications**			
**Statins**	NLM:ATC:C10AA	35% (20,657)	12% (29,810)
**Fibrates**	NLM:ATC:C10AB	3% (1,646)	1% (1,955)
**Niacin**	RXNORM:7393	2% (989)	1% (1,741)
**Omega-3 fatty acids**	RXNORM:4301	5% (2,814)	2% (5,085)

Abbreviations: ICD-10-CM, International Classification of Diseases Tenth Revision Clinical Modification, MTX, methotrexate, MetS, metabolic syndrome

Supplementary Table 3. Baseline characteristics when comparing individuals using methotrexate (MTX) and with a prior history of metabolic syndrome (MetS) and Metabolic dysfunction–Associated Liver Disease (MASLD) (MTX-MetS-MASLD cohort, n=4654) compared to those without MetS (Controls, n=263,834). Propensity score matching resulted in 4625 matched pairs

	**BEFORE PROPENSITY MATCHING**			**AFTER PROPENSITY MATCHING**		
**Baseline characteristics**	MTX-MetS-MASLD	Controls	*p*	MTX-MetS-MASLD	Controls	*p*
**Age (Mean ± SD)**	57.9 ± 13.0	53.4 ± 18.0	<0.001	57.9 ± 13.0	58.4 ± 14.3	0.047
**Sex Female**	75.1% (3487)	73.8% (191128)	0.041	75.1% (3473)	75.3% (3481)	0.847
**Race / Ethnicity**						
**Non-Hispanic White**	68.0% (3156)	65.7% (170143)	0.001	68% (3145)	69.7% (3223)	0.080
**Black or African American**	14.0% (649)	13.0% (33748)	0.056	14.0% (646)	13.1% (607)	0.236
**Hispanic or Latino**	11.8% (550)	7.6% (19804)	<0.001	11.8% (546)	10.8% (500)	0.131
**Etiology for MTX use**						
**Seropositive RA**	15.5% (721)	4.9% (12570)	<0.001	15.3% (707)	15.0% (692)	0.663
**Seronegative RA**	8.6% (401)	1.8% (4535)	<0.001	8.4% (388)	7.8% (361)	0.303
**Juvenile idiopathic arthritis**	1.0% (46)	1.2% (2982)	0.310	1.0% (46)	0.7% (33)	0.142

**Psoriasis**	18.8% (871)	6.4% (16587)	<0.001	18.6% (860)	18.4% (852)	0.830
**Connective tissue diseases**	20.8% (967)	8.7% (22509)	<0.001	20.7% (957)	20.6% (951)	0.877
**Hematopoietic malignancy**	4.3% (201)	1.6% (4200)	<0.001	4.3% (201)	4.5% (208)	0.723
**Any malignancy**	47.2% (2194)	14.6% (37717)	<0.001	47.0% (2176)	50.4% (2329)	0.001
**Glucocorticoid therapy**	84.5% (3926)	40.4% (104724)	<0.001	84.5% (3907)	84.4% (3902)	0.886
**SDHOs**	4.6% (214)	1.0% (2462)	<0.001	4.5% (210)	3.9% (181)	0.134

Abbreviations: BMI, Body Mass Index, MetS, metabolic syndrome, MTX, methotrexate, RA, rheumatoid arthritis, SD, standard deviation, SDHOs, social determinants of adverse health outcomes.

Supplementary Table 4. Baseline characteristics when comparing individuals using methotrexate (MTX) and with a prior history of metabolic syndrome (MetS) without Metabolic dysfunction–Associated Liver Disease (MASLD) (MTX-MetS-nonMASLD cohort, n=54,638) compared to those without MetS (Controls, n=263,834). Propensity score matching resulted in 54,638 matched pairs

	**BEFORE PROPENSITY MATCHING**			**AFTER PROPENSITY MATCHING**		
**Baseline characteristics**	MTX-MetS-nonMASLD	Controls	*p*	MTX-MetS-nonMASLD	Controls	*p*
**Age (Mean ± SD)**	60.8 ± 13.6	53.4 ± 18.0	<0.001	60.8 ± 13.6	61.0 ± 13.7	0.029

**Sex Female**	68.0% (37,175)	73.8% (191,128)	<0.001	68.0% (37,175)	68.1% (37,189)	0.928
**Race / Ethnicity**						
**Non-Hispanic White**	63.8% (34,844)	65.7% (170,143)	<0.001	63.8% (34,844)	63.9% (34,888)	0.782
**Black or African American**	17.4% (9,504)	13.0% (33,748)	<0.001	17.4% (9,504)	17.3% (9,462)	0.737
**Hispanic or Latino**	6.9% (3,797)	7.6% (19,804)	<0.001	6.9% (3,797)	7.0% (3,801)	0.962
**Etiology for MTX use**						
**Seropositive RA**	6.8% (3,736)	4.9% (12,570)	<0.001	6.8% (3,736)	6.5% (3,568)	0.042
**Seronegative RA**	2.5% (1,392)	1.8% (4,535)	<0.001	2.5% (1,392)	2.3% (1,239)	0.003
**Juvenile idiopathic arthritis**	0.4% (225)	1.2% (2,982)	<0.001	0.4% (225)	0.3% (191)	0.095
**Psoriasis**	8.4% (4,616)	6.4% (16,587)	<0.001	8.4% (4,616)	8.2% (4,487)	0.158
**Connective tissue diseases**	9.8% (5,338)	8.7% (22,509)	<0.001	9.8% (5,338)	9.5% (5,205)	0.173
**Hematopoietic malignancy**	2.2% (1,228)	1.6% (4,200)	<0.001	2.2% (1,228)	2.3% (1,232)	0.935
**Any malignancy**	20.5% (11,201)	14.6% (37,717)	<0.001	20.5% (11,201)	20.6% (11,259)	0.664
**Glucocorticoid therapy**	52.1% (28,462)	40.4% (104,724)	<0.001	52.1% (28,462)	51.7% (28,239)	0.177
**SDHOs**	1.3% (725)	1.0% (2,462)	<0.001	1.3% (725)	1.3% (691)	0.363

Abbreviations: BMI, Body Mass Index, MetS, metabolic syndrome, MTX, methotrexate, RA, rheumatoid arthritis, SD, standard deviation, SDHOs, social determinants of adverse health outcomes.

Supplementary Table 5. Propensity score matching analysis comparing the 3-, 5-, and 10-year outcomes of patients on methotrexate (MTX), and with a history of metabolic syndrome (MetS) and Metabolic dysfunction–Associated Liver Disease (MASLD) (MTX-MetS-MASLD cohort, n=4654 compared to those without MetS (Controls, n=263,834). Due to scarcity of cases, we were unable to run the analysis for LT incidence (≤ 10 cases in both groups). Patients with pre-inclusion outcomes were excluded from the analysis

**Outcomes**	**3-year Incidence % (N)**<br><br>MTX-MetS-MASLD vs. Controls	**5-year Incidence % (N)**<br><br>MTX-MetS-MASLD vs. Controls	**10-year Incidence % (N)**<br><br>MTX-MetS-MASLD vs. Controls	**Propensity score matching analysis (10-year outcomes)**<br><br>aOR* [95%CI]
**Liver-related outcomes**				
**Hepatic enzyme elevations**[1]	18.4% (614) vs. 13.9% (636)	21.5% (716) vs. 16.9% (776)	23.9% (797) vs. 19.4% (888)	1.31 [1.17 – 1.46]
**Hyperbilirubinemia**[2]	3.6% (162) vs. 1.8% (85)	4.5% (198) vs. 2.4% (113)	4.9% (218) vs. 3.2% (147)	1.57 [1.27 – 1.95]
**Prolonged INR**[3]	3.7% (165) vs. 2.1% (99)	4.4% (198) vs. 2.8% (128)	5.0% (226) vs. 3.4% (156)	1.52 [1.24 -1.88]
**Clinically significant DILI**[4]	4.5% (199) vs. 2.7% (126)	5.3% (233) vs. 3.3% (151)	6.0% (261) vs. 3.9% (180)	1.56 [1.28 – 1.89]
**Liver cirrhosis**	3.2% (139) vs. 1.3% (59)	3.9% (170) vs. 1.6% (73)	4.4% (190) vs. 2.0% (92)	2.21 [1.72 – 2.84]
**Other outcomes**				

**All-cause mortality**	4.6% (212) vs. 4.5% (209)	5.5% (252) vs. 5.9% (273)	6.7% (306) vs. 7.5% (346)	0.88 [0.75 – 1.03]
**All cause hospitalizations**	12.2% (422) vs. 9.0% (342)	15.1% (521) vs. 11.3% (432)	17.5% (605) vs. 13.5% (515)	1.36 [1.20 – 1.54]
**All-cause ICU admissions**	4.8% (210) vs. 3.1% (138)	5.9% (259) vs. 4.0% (181)	7.1% (309) vs. 5.0% (222)	1.46 [1.22 – 1.75]

Abbreviations: aOR, adjusted odds ratio, DILI, drug-induced liver injury, INR, international normalized ratio, MTX, methotrexate, MetS, metabolic syndrome.

**Adjusted for demographics, comorbid conditions and treatment.*

1: Hepatic enzyme elevations is defined as elevated alanine aminotransferase (ALT) > 40 U/L, aspartate aminotransferase (AST) > 40 U/L, or alkaline phosphatase (ALP) > 130 U/L

2: Hyperbilirubinemia is defined as a serum bilirubin level > 1.2 mg/dL

3: Prolonged INR is defined as an INR > 1.2

4: Clinically significant DILI is defined as ALT or AST > 200 U/L, ALP > 200 U/L, serum bilirubin > 2.5 mg/dL, and International Normalized Ratio (INR) > 1.5 or International Classification of Diseases, Tenth Revision, Clinical Modification (ICD-10-CM) codes for toxic liver injury or acute liver failure.

Supplementary Table 6. Propensity score matching analysis comparing the 3-, 5-, and 10-year outcomes of patients on methotrexate (MTX) with metabolic syndrome (MetS) and without Metabolic dysfunction–Associated Liver Disease (MASLD) (MTX-MetS-nonMASLD cohort, n=27,336) compared to those without MetS (Controls, n=27,336). Due to scarcity of cases, we were unable to run the analysis for LT incidence (≤ 10 cases in both groups). Patients with pre-inclusion outcomes were excluded from the analysis

**Outcomes**	**3-year Incidence % (N)**<br>MTX-MetS-nonMASLD vs. Controls	**5-year Incidence % (N)**<br>MTX-MetS-nonMASLD vs. Controls	**10-year Incidence % (N)**<br>MTX-MetS-nonMASLD vs. Controls	**Propensity score matching analysis (10-year outcomes)**<br>aOR* [95%CI]
**Liver-related outcomes**				
**Hepatic enzyme elevations**[1]	12.4% (6,666) vs. 9.9% (5,469)	15.7% (8,480) vs. 12.5% (6,865)	19.1% (10,223) vs. 15.1% (8,214)	1.33 [1.29 – 1.38]
**Hyperbilirubinemia**[2]	3.1% (1,706) vs. 2.1% (1,138)	3.0% (1,659) vs. 2.3% (1,274)	3.9% (2,104) vs 3.0% (1,629)	1.31 [1.22 – 1.40]
**Prolonged INR**[3]	2.3% (1,269) vs. 1.7% (962)	4.1% (2,253) vs. 2.7% (1,504)	5.3% (2,859) vs. 3.5% (1,898)	1.54 [1.45 – 1.63]
**Clinically significant DILI**[4]	2.9% (1,588) vs. 2.1% (1,172)	3.9% (2,113) vs. 2.8% (1,566)	5.0% (2,714) vs. 3.6% (1,956)	1.41 [1.33 – 1.50]
**Liver cirrhosis**	0.9% (469) vs. 0.7% (395)	3.9% (2,113) vs. 2.8% (1,566)	1.4% (766) vs. 1.2% (629)	1.22 [1.10 – 1.36]
**Other outcomes**				
**All-cause mortality**	4.6% (2,514) vs. 4.1% (2,236)	6.4% (3,516) vs. 5.7% (3,114)	8.6% (4,712) vs. 7.7% (4,172)	1.14 [1.09 – 1.19]

**All cause hospitalizations**	10.2% (5,017) vs. 7.3% (3,659)	13.1% (6,473) vs. 9.5% (4,754)	16.5% (8,068) vs. 11.9% (5,931)	1.46 [1.41 – 1.52]
**All-cause ICU admissions**	3.5% (1,887) vs. 2.3% (1,239)	4.6% (2,504) vs. 3.1% (1,661)	6.1% (3,292) vs. 4.0% (2,165)	1.56 [1.48 – 1.65]

Abbreviations: aOR, adjusted odds ratio, DILI, drug-induced liver injury, INR, international normalized ratio, MTX, methotrexate, MetS, metabolic syndrome.
**Adjusted for demographics, comorbid conditions and treatment.*
1: Hepatic enzyme elevations is defined as elevated alanine aminotransferase (ALT) > 40 U/L, aspartate aminotransferase (AST) > 40 U/L, or alkaline phosphatase (ALP) > 130 U/L
2: Hyperbilirubinemia is defined as a serum bilirubin level > 1.2 mg/dL
3: Prolonged INR is defined as an INR > 1.2
4: Clinically significant DILI is defined as ALT or AST > 200 U/L, ALP > 200 U/L, serum bilirubin > 2.5 mg/dL, and International Normalized Ratio (INR) > 1.5 or ICD-10-CM codes for toxic liver injury or acute liver failure.

Supplementary Table 7. Propensity score matching analysis comparing the 3-, 5-, and 10-year outcomes of patients on methotrexate (MTX) with metabolic syndrome (MetS) compared to those without MetS. Due to scarcity of cases, we were unable to run the analysis for LT and HCC incidence (≤ 10 cases in both groups). Patients with pre-inclusion outcomes were excluded from the analysis

	**Propensity score matching analysis** aOR* [95%CI]								
	**MTX-MetS vs. Controls**			**MTX-MetS-MASLD vs. Controls**			**MTX-MetS-nonMASLD vs. Controls**		
	**3 years**	**5 years**	**10 years**	**3 years**	**5 years**	**10 years**	**3 years**	**5 years**	**10 years**

**Liver-related outcomes**									
**Hepatic enzyme elevations[1]**	1.34 [1.30 – 1.39]	1.38 [1.34 – 1.43]	1.41 [1.38 – 1.46]	1.40 [1.24 – 1.58]	1.34 [1.12 – 1.50]	1.31 [1.17 – 1.46]	1.28 [1.23 – 1.33]	1.31 [1.27 – 1.35]	1.33 [1.29 – 1.38]
**Hyperbilirubinemia[2]**	1.41 [1.30 – 1.53]	1.41 [1.32 – 1.52]	1.40 [1.32 – 1.49]	2.02 [1.55 – 2.64]	1.86 [1.47 – 2.36]	1.57 [1.27 – 1.95]	1.33 [1.22 – 1.45]	1.32 [1.22 – 1.42]	1.31 [1.22 – 1.40]
**Prolonged INR[3]**	1.56 [1.45 – 1.68]	1.56 [1.46 – 1.66]	1.58 [1.49 – 1.67]	1.75 [1.36 – 2.25]	1.63 [1.30 – 2.04]	1.52 [1.24 - 1.88]	1.52 [1.41 – 1.64]	1.53 [1.43 – 1.63]	1.54 [1.45 – 1.63]
**Clinically significant DILI[4]**	1.45 [1.35 – 1.56]	1.45 [1.36 – 1.55]	1.49 [1.41 – 1.57]	1.69 [1.35 – 2.13]	1.66 [1.35 – 2.05]	1.56 [1.28 – 1.89]	1.37 [1.27 – 1.48]	1.37 [1.28 – 1.46]	1.41 [1.33 – 1.50]
**Liver cirrhosis**	1.41 [1.25 – 1.59]	1.50 [1.35 – 1.67]	1.48 [1.35 – 1.63]	2.51 [1.84 – 3.41]	2.49 [1.87 – 3.29]	2.21 [1.72 – 2.84]	1.19 [1.04 – 1.36]	1.25 [1.11 – 1.41]	1.22 [1.10 – 1.36]
**Other outcomes**									
**All-cause mortality**	1.11 [1.05 – 1.18]	1.13 [1.08 – 1.19]	1.13 [1.08 – 1.18]	1.02 [0.84 – 1.24]	0.92 [0.77 – 1.10]	0.88 [0.75 – 1.03]	1.13 [1.07 – 1.20]	1.14 [1.08 – 1.20]	1.14 [1.09 – 1.19]
**All cause hospitalizations**	1.47 [1.41 – 1.54]	1.49 [1.43 – 1.54]	1.43 [1.39 – 1.47]	1.41 [1.22 – 1.64]	1.39 [1.21 – 1.59]	1.36 [1.20 – 1.54]	1.44 [1.38 – 1.51]	1.45 [1.39 – 1.50]	1.46 [1.41 – 1.52]
**All-cause ICU admissions**	1.59 [1.48 – 1.70]	1.58 [1.49 – 1.68]	1.60 [1.52 – 1.69]	1.59 [1.28 – 1.98]	1.49 [1.23 – 1.82]	1.46 [1.22 – 1.75]	1.55 [1.44 – 1.67]	1.54 [1.45 – 1.64]	1.56 [1.48 – 1.65]

Abbreviations: aOR, adjusted odds ratio, DILI, drug-induced liver injury, INR, international normalized ratio, MTX, methotrexate, MetS, metabolic syndrome.

**Adjusted for demographics, comorbid conditions and treatment.*

1: Hepatic enzyme elevations is defined as elevated alanine aminotransferase (ALT) > 40 U/L, aspartate aminotransferase (AST) > 40 U/L, or alkaline phosphatase (ALP) > 130 U/L

2: Hyperbilirubinemia is defined as a serum bilirubin level > 1.2 mg/dL

3: Prolonged INR is defined as an INR > 1.2
4: Clinically significant DILI is defined as ALT or AST > 200 U/L, ALP > 200 U/L, serum bilirubin > 2.5 mg/dL, and International Normalized Ratio (INR) > 1.5 or ICD-10-CM codes for toxic liver injury or acute liver failure.

Supplementary Table 8. STROBE Statement

	Item No	Recommendation	Page No
**Title and abstract**	1	(*a*) Indicate the study's design with a commonly used term in the title or the abstract	1-2
		(*b*) Provide in the abstract an informative and balanced summary of what was done and what was found	
**Introduction**			
Background/rationale	2	Explain the scientific background and rationale for the investigation being reported	3
Objectives	3	State specific objectives, including any prespecified hypotheses	4-5
**Methods**			
Study design	4	Present key elements of study design early in the paper	6
Setting	5	Describe the setting, locations, and relevant dates, including periods of recruitment, exposure, follow-up, and data collection	6

Participants	6	(*a*) Give the eligibility criteria, and the sources and methods of selection of participants. Describe methods of follow-up	7-9
		(*b*) For matched studies, give matching criteria and number of exposed and unexposed	9-10
Variables	7	Clearly define all outcomes, exposures, predictors, potential confounders, and effect modifiers. Give diagnostic criteria, if applicable	9-10
Data sources/ measurement	8*	For each variable of interest, give sources of data and details of methods of assessment (measurement). Describe comparability of assessment methods if there is more than one group	7-9
Bias	9	Describe any efforts to address potential sources of bias	9
Study size	10	Explain how the study size was arrived at	8
Quantitative variables	11	Explain how quantitative variables were handled in the analyses. If applicable, describe which groupings were chosen and why	11
Statistical methods	12	(*a*) Describe all statistical methods, including those used to control for confounding	11-12
		(*b*) Describe any methods used to examine subgroups and interactions	11-12
		(*c*) Explain how missing data were addressed	
		(*d*) If applicable, explain how loss to follow-up was addressed	
		(*e*) Describe any sensitivity analyses	
**Results**			

Participants	13*	(a) Report numbers of individuals at each stage of study—eg numbers potentially eligible, examined for eligibility, confirmed eligible, included in the study, completing follow-up, and analyzed	8
		(b) Give reasons for non-participation at each stage	
		(c) Consider use of a flow diagram	
Descriptive data	14*	(a) Give characteristics of study participants (eg demographic, clinical, social) and information on exposures and potential confounders	8, 9
		(b) Indicate number of participants with missing data for each variable of interest	
		(c) Summarize follow-up time (eg, average and total amount)	
Outcome data	15*	Report numbers of outcome events or summary measures over time	13
Main results	16	(*a*) Give unadjusted estimates and, if applicable, confounder-adjusted estimates and their precision (eg, 95% confidence interval). Make clear which confounders were adjusted for and why they were included	14-15
		(*b*) Report category boundaries when continuous variables were categorized	
		(*c*) If relevant, consider translating estimates of relative risk into absolute risk for a meaningful time period	
Other analyses	17	Report other analyses done—eg analyses of subgroups and interactions, and sensitivity analyses	15-16
**Discussion**			
Key results	18	Summarize key results with reference to study objectives	17-18

Limitations	19	Discuss limitations of the study, taking into account sources of potential bias or imprecision. Discuss both direction and magnitude of any potential bias	20
Interpretation	20	Give a cautious overall interpretation of results considering objectives, limitations, multiplicity of analyses, results from similar studies, and other relevant evidence	17-18
Generalisability	21	Discuss the generalisability (external validity) of the study results	19
**Other information**			
Funding	22	Give the source of funding and the role of the funders for the present study and, if applicable, for the original study on which the present article is based	N/A
